# A sinogram denoising algorithm for low-dose computed tomography

**DOI:** 10.1186/s12880-016-0112-5

**Published:** 2016-01-22

**Authors:** Davood Karimi, Pierre Deman, Rabab Ward, Nancy Ford

**Affiliations:** 2366 Main Mall, Vancouver, V6T 1Z4 BC Canada; 2151 Wesbrook Mall, Vancouver, V6T 1Z3 BC Canada

**Keywords:** Computed tomography, Sinogram denoising, Poisson noise, Total variation, Low-dose CT

## Abstract

**Background:**

From the viewpoint of the patients’ health, reducing the radiation dose in computed tomography (CT) is highly desirable. However, projection measurements acquired under low-dose conditions will contain much noise. Therefore, reconstruction of high-quality images from low-dose scans requires effective denoising of the projection measurements.

**Methods:**

We propose a denoising algorithm that is based on maximizing the data likelihood and sparsity in the gradient domain. For Poisson noise, this formulation automatically leads to a locally adaptive denoising scheme. Because the resulting optimization problem is hard to solve and may also lead to artifacts, we suggest an explicitly local denoising method by adapting an existing algorithm for normally-distributed noise. We apply the proposed method on sets of simulated and real cone-beam projections and compare its performance with two other algorithms.

**Results:**

The proposed algorithm effectively suppresses the noise in simulated and real CT projections. Denoising of the projections with the proposed algorithm leads to a substantial improvement of the reconstructed image in terms of noise level, spatial resolution, and visual quality.

**Conclusion:**

The proposed algorithm can suppress very strong quantum noise in CT projections. Therefore, it can be used as an effective tool in low-dose CT.

## Background

Computed tomography (CT) is one of the most widely used imaging modalities in medicine and its usage has been consistently growing over the past decades [1]. Although CT is an indispensable tool in modern medicine, one of its drawbacks is that the radiation used in this type of imaging can be harmful to the patient health. Reducing the radiation dose requires reducing the number of projection measurements or reducing the radiation intensity, which in turn results in noisier measurements. Reconstructing a high-quality CT image from such measurement is a great challenge. The traditional image reconstruction methods in CT are based on filtered back-projection (FBP). Although FBP-based methods are fast and relatively easy to implement, they perform very poorly when the number of projections is reduced or when the measurements are very noisy. With increased awareness of the harmful effects of excessive radiation, recent years have witnessed a new interest in statistical reconstruction methods in CT. These methods promise reconstructing a high-quality image from undersampled or noisy projections. Statistical image reconstruction methods in CT are based, as their name implies, on including the statistics of the detected photon counts in the reconstruction process. They can be classified into three categories [2, 3]:

(A) Methods that work on the raw data (sinogram)- these are mostly smoothing techniques that aim at reducing the Poisson noise in the projection data. The processed sinogram data are then used to reconstruct the image using an iterative or filtered backprojection (FBP) method.
(B) Methods that work on the reconstructed image- these are also denoising techniques, but operate in the image domain.
(C) Methods that use statistical modeling of the imaging process to devise iterative algorithms for image reconstruction.

Whereas the methods in category (C) usually lead to better image quality, the first two types of methods are usually easier to implement, faster, and independent of the image reconstruction method. Among the first two categories of methods, category (A) is usually much more effective. This is because while we have a good understanding of the properties of the noise in the raw (i.e. projection) data, the statistics of the noise in the reconstructed image are not as well understood. Hence, methods that filter the reconstructed image are post-processing algorithms that cannot use the photon statistics and their performance is in general inferior to filtering in the raw data domain.

Previous studies in sinogram denoising are numerous and they present a diverse set of possible approaches to the problem. Because the variance of the Poisson noise changes with the signal amplitude, adaptive filters are a natural choice and they have been researched extensively [4, 5]. Bayesian approaches, usually maximizing a weighted sum of the data likelihood and a regularity term, have also been proposed in several studies [6, 7]. These algorithms can be very elaborate because the resulting optimization problem is usually hard to solve. At the other end of the spectrum of algorithmic complexity, there exist very simple methods involving shift-invariant low-pass filters that reduce the high-frequency variations in the sinogram [8, 9]. As one might expect, these methods are much less effective because they do not consider the signal-dependent nature of the noise. Another class of algorithms that have been used for sinogram denoising include multi-resolution methods involving short-time Fourier and wavelet transforms [10, 11]. These methods are based on a standard signal processing approach of separating the noise from the signal in the transform domain. A number of studies have focused on denoising the projection data in the Radon space (i.e. after the logarithm transformation). Even though the photon counts follow a Poisson distribution, the noise in the Radon space follows an approximately Gaussian distribution [12, 13]. Therefore, some studies have proposed algorithms for noise suppression in the Radon space based on the assumption that the noise is normally distributed [14, 15]. The Gaussianity assumption significantly simplifies the problem and widens the range of tools that can be employed in algorithm development. However, as the photon counts decrease, the distribution of the noise in the Radon space can significantly depart from a Gaussian distribution [13]. Therefore, these methods become less efficient in low-dose setting, which is exactly where noise suppression is mostly needed.

In the past decade, image denoising has witnessed a growing interest in patch-based methods. The two main classes of patch-based denoising methods include dictionary-based denoising methods and nonlocal-means methods. Dictionary-based denoising methods are based on the assumption that small patches of natural images have a sparse representation in a (usually overcomplete) dictionary that can be learned from training data [16, 17]. As opposed to more traditional denoising methods that use an off-the-shelf basis (e.g., a wavelet basis), these dictionary-based methods adapt the dictionary to the specific class of images at hand. If the dictionary is well-trained, genuine image features will have a sparse representation in the dictionary, whereas noise will not have such a sparse representation. Therefore, sparse representation of image patches will lead to denoising. Dictionary-based denoising methods have shown to be highly successful in denoising of general natural images [16, 17] as well as medical images [18, 19]. The nonlocal-means methods, on the other hand, exploit the self-similarities in natural images. To estimate the denoised value of each pixel, they consider a small patch centered on that pixel and search the image for patches that are similar to this patch. The true (i.e., denoised) value of the pixel is estimated by some collaborative filtering of the found similar patches. This collaborative filtering was in the form of a weighted averaging in the original nonlocal-means algorithm [20, 21], but has much more elaborate forms in more recent algorithms [22]. Although the origins of these denoising methods go back approximately 10 years, they have been extended and improved in many ways and they present some of the best available image denoising methods. One of the limitations of patch-based methods, especially for large 3D images that are common in medical applications, is their high computational demands.

In this study, we suggest smoothing the noisy sinograms by minimizing a cost function that consists of a data likelihood term and a regularization term that promotes gradient sparsity. The resulting optimality condition suggests an adaptive filter that carries out a stronger denoising where the signal intensity is higher, which is the expected outcome under Poisson distribution. Instead of solving the resulting optimization problem directly, we suggest modifying an existing algorithm such that it approximates the exact solution locally. Therefore, our approach computes the denoised value of each sinogram pixel by solving a local optimization problem in a small neighborhood around that pixel. We will evaluate the performance of the suggested approach by applying it to noisy simulated projections and low-dose projections acquired from a micro-CT scanner.

## Methods

Given a noisy image, *v*, the goal is to find an image *u* that ideally preserves all important features in the image while reducing the unwanted noise. Since this is an ill-posed inverse problem, additional information needs to be introduced to regularize the problem. The denoised image can then be found by minimizing a cost function of the form:

(1) $$  E_{\lambda}(u)= \frac{1}{2} ||u-v||_{2}^{2} + \lambda R(u)  $$

The first term in *E*_*λ*_(*u*) reflects the requirement that the denoised image should be close to the noisy image and the second term is the regularization. In the context of denoising problem formulated as (1), a proper regularization is necessary because without a regularizing term the noisy image itself is the only solution of the problem; i.e., if *λ*=0 then *u*=*v* is the only minimizer of *E*_*λ*_(*u*). A famous example of variational denoising methods is the Rudin-Osher-Fatemi (ROF) model that has the following form [23]:

(2) $$  E_{\lambda}(u)= \frac{1}{2} ||u-v||_{2}^{2} + \lambda \int_{\Omega} |\nabla u|  $$

where *Ω* denotes the image domain and *λ* is the regularization parameter. The second term, where ∇*u* is the gradient of *u*, is the regularization term. The choice of *ℓ*_2_-norm in the first term stems from the assumption of additive Gaussian noise. For the case of Poisson distribution considered in this study, this term has to be modified.

Let *u*(*i*,*j*) and *v*(*i*,*j*) denote pixels of discretized versions of *u* and *v*. For an arbitrary pixel location (from the probability mass function of a variable with Poisson distribution):

(3) $$  P(v(i,j);u(i,j))= \frac{e^{-u(i,j)} u(i,j)^{v(i,j)}}{v(i,j)!}  $$

assuming the pixel values are independent, for the entire image we will have:

(4) $$  P(v|u)= \prod\limits_{i,j} \frac{e^{-u(i,j)} u(i,j)^{v(i,j)}}{v(i,j)!}  $$

We ignore the denominator, which is independent of *u*. Since we want to find a functional to minimize, we consider the negative logarithm of the numerator:

(5) $$  -\text{log}(P(v|u) \propto \sum\limits_{i,j} u(i,j)- v(i,j) \text{log}\,(u(i,j))  $$

With this modified fidelity term, the new energy functional to be minimized will have the following form [24]:

(6) $$  E_{\lambda}(u)= \frac{1}{2} \int_{\Omega} (u-v \, \text{log} u) + \lambda \int_{\Omega} |\nabla u|  $$

Now, compare the optimality conditions for the two models (obtained from the Euler-Lagrange equation):

(7) $$  {}\left\{ \begin{array}{ll} (u - v) + \lambda \, p =0 & \quad \text{For the ROF model in (2)}\\ (u - v) + (\lambda u) \, p =0 & \quad \text{For the new model in (6)} \end{array} \right.  $$

where *p* is a sub-gradient of $\int _{\Omega } |\nabla u|$. The only difference between the two equations is the dependence of the regularization parameter on *u* in the new model. The new model suggests that a stronger smoothing must be applied where signal has larger values. This outcome agrees with what we expect since under Poisson distribution noise level is proportional to signal intensity.

Minimization of the new functional in (6) is not a straightforward problem. One approach is to first replace |∇*u*| with $\sqrt {|\nabla u|^{2}+\epsilon }$ for a small *ε*>0 and then to apply a gradient descent iteration [24]. Another approach, suggested in [25], is to use a Taylor’s expansion of the data fidelity term and to minimize this approximate model. In this paper, we follow an algorithm that was developed for the original ROF model [26]. However, we denoise each sinogram pixel separately by minimizing *E*_*λ*_(*u*) in a small neighborhood around that pixel, and with a regularization parameter inspired by the new model.

Total variation denoising has proved to be very effective when applied to piecewise-constant images. On more complex images, however, it can lead to undesired effects. Most importantly, on images with piecewise-linear features, i.e. regions with smooth change in intensity, ROF’s original model leads to staircase artifacts. This is because, by design, ROF’s model favors piecewise-constant solutions. We believe that this can be a major problem when applying TV denoising to sinogram images because even if the imaged object is piecewise-constant, its projections can be very far from piecewise-constant. This is easy to visualize because a feature with uniform intensity in the imaged object will have a piecewise-constant projection in the sinogram only if different rays from the x-ray source to the detector plane cut the same length through that feature, which is a very unlikely scenario. Hence, the projections are very likely to contain features of higher orders of regularity (i.e., piecewise-linear, piecewise-quadratic, etc.) that would suffer from a staircase effect when treated with the ROF model. A heavily researched approach to reducing the staircase effect is to replace the *ℓ*_1_ norm of the gradient with the *ℓ*_1_ norm of higher-order differential operators [27, 28]. A less sophisticated approach, but one that has a trivial implementation, is to perform the total variation minimization locally. This approach has also been shown to alleviate the staircase effect [29]. Moreover, with a local minimization strategy, if the size of the neighborhood considered in minimization is small enough, one can safely assume that the signal intensity and noise level are approximately constant. Therefore, a solution based on the ROF’s original model will be a good approximation to the solution of the new model based on Poisson noise. This way, we can utilize efficient existing algorithms for the ROF model while avoiding the staircase artifacts.

Since our approach is based on Chambolle’s famous algorithm [26], we briefly describe this algorithm here. If we denote by *X* and *Y* the space of the image *u* and its gradient, ∇*u*, respectively, then an alternative definition of total variation of *u* is:

(8) $$  \sum\limits_{i,j} |\nabla u|_{i,j}= \text{sup} \left\{ \langle p,\nabla u \rangle_{Y} : p \in Y, |p_{i,j}| \leq 1 \right\}  $$

Chambolle introduced the discrete divergence operator as the dual of the gradient operator, i.e. 〈*p*,∇*u*〉_*Y*_=〈−div *p*,*u*〉_*X*_. In the discrete image domain:

(9) $$  \left(\text{div} \, p\right)_{i,j}= \left(p_{i,j}^{1}-p_{i-1,j}^{1}\right)+ \left(p_{i,j}^{2}-p_{i,j-1}^{2}\right)  $$

Because of the duality of the gradient and divergence operators, total variation can also be written as:

(10) $$  {}\sum\limits_{i,j} |\nabla u|_{i,j}= \mathop{\text{sup}}_{z \in K} \, \langle z,u \rangle_{X} \; \; \; \; K= \{\text{div }p: p \in Y, \, |p_{i,j}| \leq 1 \}  $$

The minimizer of the energy functional in (2) is then obtained by projecting *v* onto the set *λK*:

(11) $$ u= \, v- \pi_{\lambda K}(v)  $$

which is equivalent to minimizing the Euclidian distance between *v* and *λ* div *p*, and this can be achieved via the following iteration for computing *p*:

(12) $$  p^{0}=0; \,\,\, p_{i,j}^{n+1}= \frac{p_{i,j}^{n}+\tau \left(\nabla(\text{div} \, p^{n} -v/\lambda)\right)_{i,j}}{1+\tau |\left(\nabla(\text{div} \, p^{n} -v/\lambda)\right)_{i,j}|}  $$

where *τ*>0 is the step size. For a small enough step size, *τ*≤1/8, the algorithm is guaranteed to converge [26].

Instead of a global solution, we minimize the energy functional (6) in a small neighborhood of each pixel. To this end, let us denote by *ω* the set of indices that define the desired neighborhood around the current pixel. For example, for a square neighborhood of size (2*m*+1)×(2*m*+1) pixels that we used in this study, $\omega =\left \lbrace (i,j): \, i,j=-m:m \right \rbrace $. We also consider a normalized Gaussian weighting function on this neighborhood:

(13) $$  W(i,j)= \exp \left(-\frac{(i^{2}+j^{2})}{h^{2}}\right)  $$

The local problem will then become that of minimizing the following functional:

(14) $$  E_{\lambda,W}(u')= \frac{1}{2} ||u'-v_{\omega}||_{W}^{2} + \lambda' \int_{\omega} |\nabla u'|  $$

where $||.||_{W}^{2}$ denotes the weighted norm with weights *W* and *v*_*ω*_ denotes the noisy image restricted to the window *ω* around the current pixel. It must be clear that here *u*^′^ is a function defined only on the neighborhood *ω*. The solution of this local optimization problem will be similar to Chambolle’s algorithm described above [29]. The only difference is in the update formula for *p*:

(15) $$  p^{0}=0; \,\,\, p_{i,j}^{n+1}= \frac{p_{i,j}^{n}+\tau \left(\nabla\left(D^{-1}\text{div} \, p^{n} -v/\lambda'\right)\right)_{i,j}}{1+\tau |\left(\nabla\left(D^{-1} \text{div} \, p^{n} -v/\lambda'\right)\right)_{i,j}|}  $$

where *D* is a diagonal matrix whose diagonal elements are the values of *W*.

The regularization parameter, *λ*^′^, must be chosen according to (7). The simplest approach is to set *λ*^′^= *λv*(*i*,*j*), where *λ* is a global regularization parameter and *v*(*i*,*j*) is the value of the current pixel in the noisy image. Since *v*(*i*,*j*) is noisy, a better choice is to use a weighted local average as the estimate of the intensity of the true image at the current pixel (note that the maximum-likelihood estimate of the mean of a Poisson process from a set of observations is the arithmetic mean of the observations). Therefore, we suggest the following choice for the local regularization parameter.

(16) $$  {} \begin{aligned} \lambda'&= \lambda \, \frac{\sum_{-a \leq i',j' \leq a} W'\left(i',j'\right)v\left(i-i',j-j'\right)}{\sum_{-a \leq i',j' \leq a}W'\left(i',j'\right)}\quad \text{where}\\ W'(i,j)&= \exp \left(-\frac{\left(i^{2}+j^{2}\right)}{h'^{2}}\right) \end{aligned}  $$

There are several parameters in the proposed algorithm. The global regularization parameter *λ* controls the strength of the denoising. It should be set based on the desired level of smoothing. Parameter *m* sets the size of the neighborhood considered around each pixel, which in this study was chosen to be a square window of size (2*m*+1)×(2*m*+1). Numerical experiments in [29] have shown that in total variation denoising, the influence map of a pixel is usually limited to a radius of approximately 10 pixels for typical values of the regularization parameter. Therefore, a good value for *m* would be around 10, which is the value we used for all experiments reported in this paper. The width of the Gaussian weighting function *W* is adjusted through *h*. We used *h*=2*m* which we found empirically to work well. Similarly, *a* and *h*^′^ in (16) determine the size of the window and the weights used for determining the local regularization parameter. These have to be set based on the noise level in the image; larger values should be chosen when noise is stronger. In this study, we used *a*=4 and *h*^′^=2*a*.

A simple implementation of the proposed algorithm can be computationally intensive because it will involve solving a minimization problem, though very small, for every individual pixel in the sinogram. This will be a major drawback because a big advantage of sinogram denoising methods, compared to iterative image reconstruction methods, is the shorter computational time. To reduce the computational time, after minimizing the local cost function (14) around the current pixel, we will replace the value of all pixels in the window of size (2*a*+1)×(2*a*+1) around the current pixel, instead of just the center pixel, and then shift the window by (2*a*+1). Our extensive numerical experiments with simulated and real projections showed that with this approach the results will be almost identical to the case where only one pixel is denoised at a time. This is the approach that we followed in all experiments reported in this paper.

## Results and discussion

We evaluated the proposed denoising algorithm on simulated projections and two sets of real low-dose projections of a physical phantom and a rat obtained using a micro-CT scanner. We compared the performance of our proposed algorithm with two other methods:

1. A bilateral filtering algorithm proposed in [14]. This bilateral filtering algorithm works by minimizing a cost function of the following form:
  $$ E(u)= \sum\limits_{k} \sum\limits_{k' \in \Omega_{k}} P_{1}(k,k') P_{2}(k,k') $$
  where *k* denotes the index of a pixel, *Ω*_*k*_ is a neighborhood around this pixel, and *P*_1_ and *P*_2_ are two cost functions in terms of the spatial distance and difference in pixel values, respectively, both having Gaussian forms:
  $$ P_{1}(k,k')= \exp \left(- \frac{\left(k-k'\right)^{2}}{2 d^{2}} \right) $$
  $$ P_{2}(k,k')= 1- \exp \left(- \frac{\left(u_{k}-u_{k'}\right)^{2}}{2 \sigma^{2}} \right) $$
  The algorithm parameters include *d* and *σ* that control the range of weighting for *P*_1_ and *P*_2_. The authors in [14] suggest a fixed filter length of *w*=5 and fixed *d*=*w*/6=5/6 (that they found empirically to be a good choice) and try a range of values for *σ* between 0.7 and 2.8. In this study, we applied the bilateral filtering for several values of *σ* in this range and apply the proposed algorithm for several values of the regularization parameter, *λ*.
2. A nonlocal principal component analysis (NL-PCA) algorithm proposed in [30]. In this method, patches of the image are first clustered using the K-Means algorithm. For all patches in a cluster a Poisson PCA (also known as exponential PCA) is performed to denoise them. The PCA problem is solved via alternating Newton’s iterations. The denoised patches are returned to their original locations and averaged (to account for the patch overlaps) in order to form the denoised image. Patch-based methods are computationally very intensive. Therefore, with this algorithm we used parameter settings that resulted in a reasonable computational time.

### Simulated data

We simulated 360 noisy cone-beam projections, from 0° to 359° with 1° increments from a 3D Shepp-Logan phantom according to the following model [12, 31]:

(17) $$  {N_{d}^{i}} = {N_{0}^{i}} \; e^{\left(-\int_{i} \mu ds \right)}  $$

where ${N_{d}^{i}}$ and ${N_{0}^{i}}$ denote, respectively, the number of detected and incident photons for the ray that extends from the source to the detector *i* and $\int _{i} \mu ds$ is the line integral of the attenuation coefficient along that ray. We assumed ${N_{0}^{i}}$ to be constant for all *i*, i.e., no bowtie filtration, and ${N_{d}^{i}}$ to be a Poisson-distributed random variable with the expected value given by (17). We used two values of ${N_{0}^{i}}= 500$ and 2000 to simulate two sets of projections, which we will call high-noise and low-noise, respectively. The phantom size was 512×512×512 voxels and the projections were each 700×700 pixels in size.

**Fig. 1:**
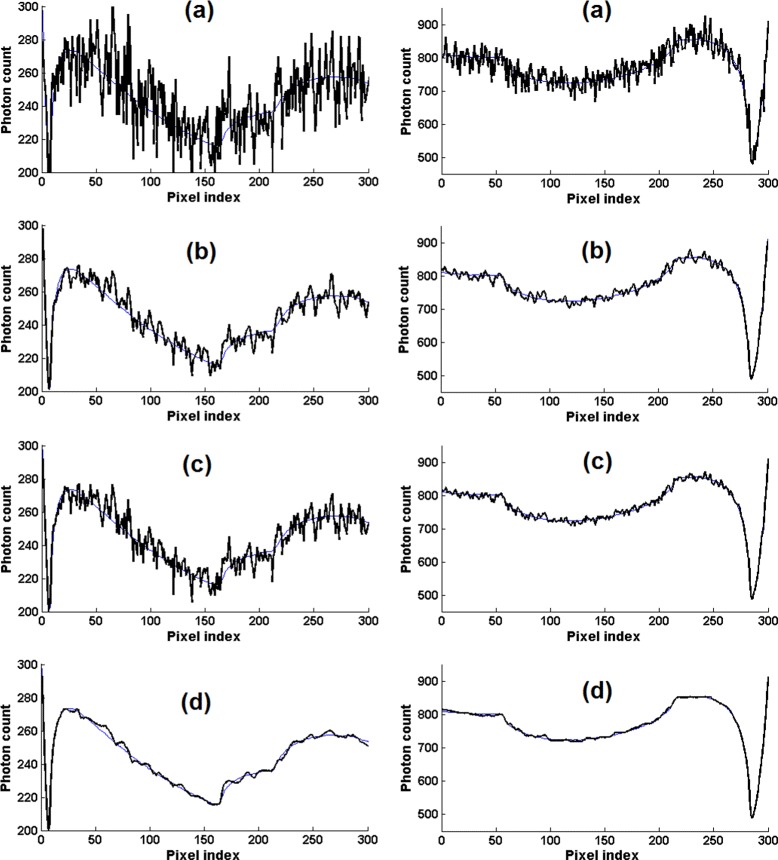
Two typical one-dimensional profiles of the noisy and denoised projections simulated from the Shepp-Logan phantom. The thin blue curve in each plot shows the corresponding noise-free projection. The left column is for the high-noise case and the right column is for the low-noise case. **a** the noisy sinogram, **b** denoised using bilateral filtering, **c** denoised with NL-PCA, and **d** denoised with the proposed TV-based algorithm
- Mutual information (MI), which treats the projections as stochastic processes [32, 33]:
  $$ \text{MI}(u^{*},\hat{u})= \sum\limits_{i=1}^{h} \sum\limits_{j=1}^{h} p(u^{*}_{i}, \hat{u}_{j}) \text{log} \left(\frac{p(u^{*}_{i}, \hat{u}_{j})}{p(u^{*}_{i}) p(\hat{u}_{j})} \right) $$
  Here, *u*^∗^ and $\hat {u}$ represent the true and denoised projections, respectively. We used histograms of *u*^∗^ and $\hat {u}$ for estimating $p(u^{*}_{i})$ and $p(\hat {u}_{j})$ and their joint histogram for estimating $p(u^{*}_{i},\hat {u}_{j})$, and *h* is the number of bins in the histograms. We normalized the computed $\text {MI}(u^{*},\hat {u})$ by dividing it by MI(*u*^∗^,*u*^∗^).

Figure 1 shows one-dimensional profiles of the noisy and denoised projections. The plots in this figure show that the proposed TV-based denoising significantly removes the noise and seems to be superior to bilateral filtering and NL-PCA. For quantitative comparison, because we have access to the true noise-free projections, we use the following criteria:

- Root Mean Square of the Error (RMSE), where error is defined as the difference between the denoised and the true (i.e., noise-free) projections.

**Fig. 2:**
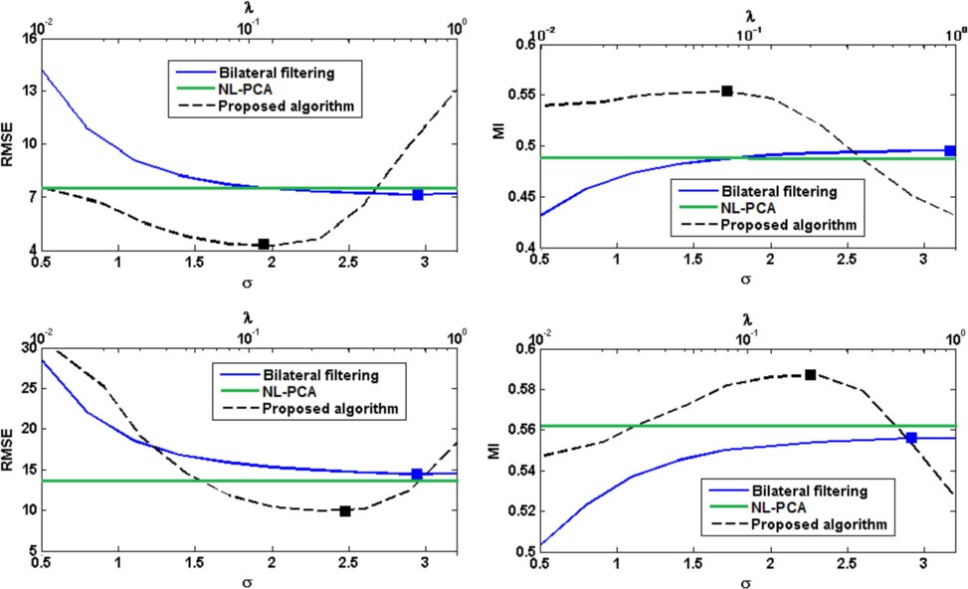
Comparison between different denoising algorithms in terms of RMSE and MI for the high-noise projections (*top row*) and low-noise projections (*bottom row*) simulated from the Shepp-Logan phantom. Values for the bilateral filtering algorithm are plotted as a function of *σ* (the *bottom horizontal axis*), whereas the values for the proposed algorithm are plotted as a function of the regularization parameter *λ* (the *top horizontal axis*). The solid squares indicate the points of optimum

Figure 2 shows the plots of RMSE and MI for the proposed algorithm, bilateral filtering, and NL-PCA. For the proposed algorithm, we have plotted these values for 10 logarithmically-spaced values of *λ* in the range [0.01,1], which we found to give the best denoising results. For bilateral filtering, following [14], we have plotted these values for 10 linearly-spaced values of *σ* in the range [0.5,3.2]. From these plots it is clear that the proposed algorithm has achieved significantly better denoising results than bilateral filtering and NL-PCA. Best results with the proposed algorithm are achieved with *λ* values around 0.1 and the denoising is too strong for *λ*>1. For bilateral filtering, we found that best denoising results were usually obtained for values of *σ* close to 3.0 and the performance did not improve or slightly deteriorated when *σ* was increased beyond 3.2. The solid squares on these plots show the optimum value of the corresponding parameter (i.e., lowest RMSE or highest MI). The phantom profiles shown in Fig. 1 for the proposed algorithm and bilateral filtering were obtained with the parameter values that resulted in the lowest RMSE.

### Real cone-beam projections of a physical phantom

Cone-beam projections were acquired from a physical phantom using a Gamma Medica eXplore CT 120 micro-CT scanner. The scanner had a flat panel detector. The distance from the source to the detector and to the axis of rotation were 449.29 *mm* and 397.04 *mm*, respectively. We generated two scans of the same phantom:

1. **Low-noise scan.** Consisting of 720 projections between 0° and 360° at 0.5° intervals. The tube voltage, tube current, and exposure time were 70 kV, 40 mA, and 25 ms, respectively.
2. **High-noise scan.** Consisting of 240 projections between 0° and 360° at 1.5° intervals. The tube voltage, tube current, and exposure time (per projection) were 50 kV, 32 mA, and 16 mAs, respectively.

Since we do not have access to the true (i.e., noise-free) projections, we compare the performance of the denoising algorithms in terms of the quality of the reconstructed images. We used the low-noise scan to reconstruct a high-quality image of the phantom using the Feldkamp-Davis-Kress (FDK) algorithm [34]. We will refer to this as “the reference image”. To evaluate the denoising algorithms, we applied them on the high-noise projections, reconstructed the image of the phantom from the denoised projections using the FDK algorithm, and compared the reconstructed image with the reference image. Similar to the experiment with the simulated projections, we performed the denoising for 10 linearly-spaced values of *σ* in the range [0.5,3.2] for bilateral filtering. Similarly, we ran the proposed algorithm with 10 logarithmically-spaced values of *λ* in the range [0.001,0.1]. Each projection was 875×568 pixels in size and the size of the reconstructed image of the phantom was 880×880×650 voxels, with isotropic voxels of 0.1×0.1×0.1 *mm*^3^.

In order to assess the overall quality of the reconstructed images, we use the following two criteria:

- Root Mean Square of the Error (RMSE). Where error is defined as the difference between the image reconstructed from denoised projections and the reference image.
- Structural similarity index (SSIM) between the reconstructed image and the reference image. SSIM is used as a measure of the overall closeness of two images and is defined as [35]:
  $$ \text{SSIM}(x,\hat{x})= \frac{\left(2\mu_{x} \mu_{\hat{x}}+C_{1}\right)\left(2\sigma_{x \hat{x}}+C_{2}\right)}{\left({\mu_{x}^{2}}+\mu_{\hat{x}}^{2}+C_{1}\right)+\left({\sigma_{x}^{2}}+\sigma_{\hat{x}}^{2}+C_{2}\right)} $$
  where *μ*_*x*_ and *σ*_*x*_ represent the mean and standard deviation of the image *x*, $\sigma _{x\hat {x}}$ is its covariance with image $\hat {x}$, and *C*_1_ and *C*_2_ are constants.

**Fig. 3:**
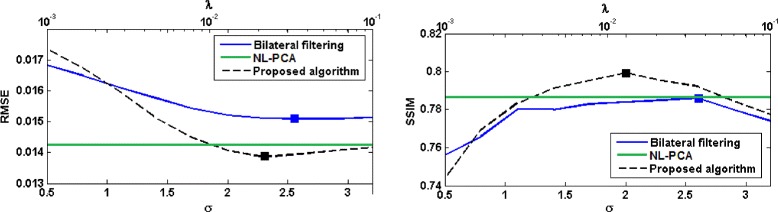
Performance comparison between different sinogram denoising algorithms in terms of RMSE and SSIM on the scan of the physical phantom. Values for the bilateral filtering algorithm are plotted as a function of *σ* (the *bottom horizontal axis*), whereas the values for the proposed algorithm are plotted as a function of the regularization parameter *λ* (the *top horizontal axis*). The solid squares indicate the points of optimum

The plots of RMSE and SSIM are shown in Fig. 3. Compared with both bilateral filtering and NL-PCA, the image reconstructed from projections denoised using the proposed algorithm has a significantly lower RSME and higher SSIM. Best results in terms of SSIM with the proposed algorithm are obtained with *λ*=0.0129 and for bilateral filtering algorithm with *σ*=2.6.

**Fig. 4:**
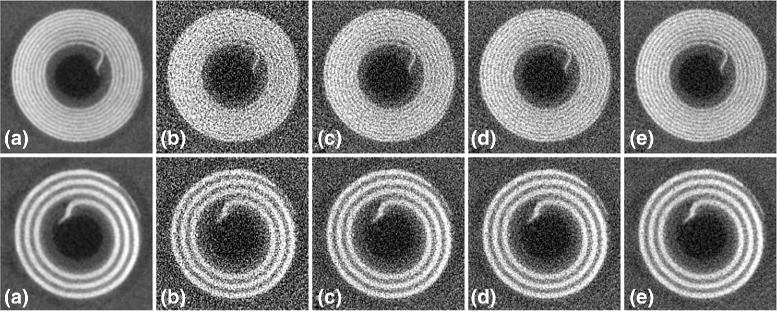
The 200−*μ* *m* (*top row*) and 500−*μ* *m* (*bottom row*) coils in the images reconstructed from noisy and denoised projections of the physical phantom; **a** the reference image, **b** without denoising, **c** bilateral filtering, **d** NL-PCA, and **e** the proposed algorithm

**Fig. 5:**
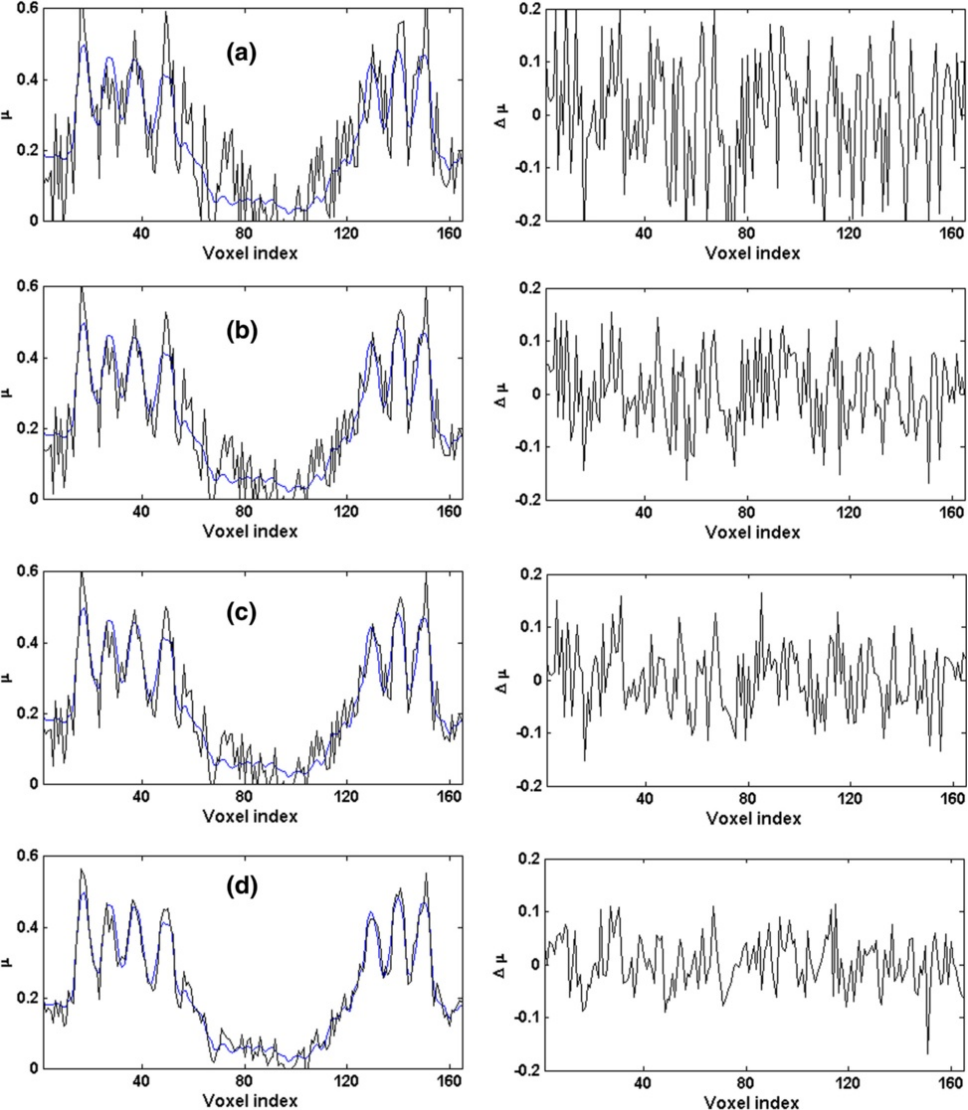
The left column shows a profile through the 500−*μ* *m* coil in the images of the physical phantom reconstructed from noisy and denoised projections: **a** without denoising, **b** bilateral filtering, **c** NL-PCA, and **d** the proposed algorithm. In each of these plots in the left column, we have included the profile of the reference image (the blue curves). For a better comparison of the denoising algorithms, in the right column we have shown the difference between the profiles shown in the left column and the profile of the reference image

The plots in Fig. 3 reflect the overall closeness of the images reconstructed from the denoised projections and the reference image. The imaged phantom had different modules that allowed for a more detailed evaluation of the quality of the reconstructed images [36]. A set of fine coils inside the phantom allow for visual inspection of the spatial resolution in the reconstructed image. Figure 4 shows two of these coils in the images reconstructed from noisy and denoised projections. These coils have thicknesses of 500 *μm* and 200 *μm*, corresponding to spatial resolutions of 1 and 2.5 line pairs per *mm*, respectively. The image shown for the proposed algorithm corresponds to *λ*=0.0129 and the image shown for bilateral filtering corresponds to *σ*=2.6. As we mentioned above, these parameter values led to highest SSIM. The images show a marked improvement in the image quality via sinogram denoising. It also seems that the proposed algorithm leads to a smoother image without affecting the spatial resolution. In Fig. 5 we have shown a profile through the center of the 500−*μm* coil for the images reconstructed from noisy and denoised projections and also the difference between them and the reference image for a closer comparison. It is clear from these profiles that the image reconstructed from the projections denoised using the proposed algorithm are closer to the reference image.

In order to compare the denoising algorithms in terms of the trade-off between noise and spatial resolution, we followed an approach similar to that suggested in [14]. Specifically, we computed the following two numbers as measures of spatial resolution and noise level in the reconstructed image of the phantom: **Measure of spatial resolution.** The imaged phantom included a slanted edge that consisted of a plastic-air boundary, specially designed for accurate estimation of the modulation transfer function (MTF). We used the method proposed in [37] for estimation of the MTF over the range of spatial frequencies between 0 and 5 *mm*^−1^. As also suggested in [14], we use the spatial frequency at which the normalized MTF reaches a value of 0.10 as a representative number for spatial resolution. **Measure of noise level.** A uniform polycarbonate disk is included in the phantom for the purpose of assessing the noise level in the reconstructed image. We selected five cubes, each 10×10×10 voxels, at different locations within this disk and computed the standard deviation of the voxel values in each cube. We use the average standard deviation of voxel values in these cubes as a measure of noise level.

**Fig. 6:**
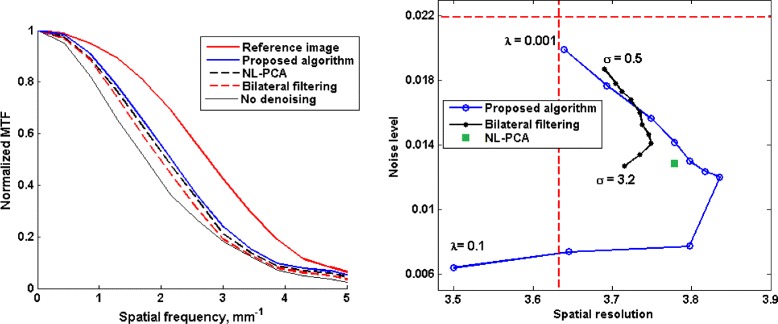
*Left*: plots of the normalized MTF obtained by different sinogram denoising algorithms and those of the reference image and the image reconstructed without sinogram denoising. *Right*: plots of noise level versus spatial resolution for different denoising algorithms. The dashed horizontal and vertical lines in this plot show the corresponding values for the image reconstructed without sinogram denoising

We computed the above two values for bilateral filtering algorithm with 10 linearly-spaced values of *σ* in the range [0.5,3.2] and for the proposed TV-based algorithm with 10 logarithmically-spaced values of *λ* in the range [0.001,0.1]. In Fig. 6, we have shown plots of these two values for the three denoising algorithms. Note that a high spatial resolution and a low noise level are desirable. Therefore, all three denoising algorithms have improved the quality of the reconstructed image for the range of parameter values used (except for *λ*=0.1 with the proposed algorithm). Moreover, the proposed algorithm has achieved better results than bilateral filtering and NL-PCA. Specifically, for *λ*∈[0.0077,0.0359] the proposed algorithm has achieved both higher spatial resolution and lower noise than bilateral filtering (for any parameter value) and NL-PCA. In Fig. 6, we have also shown plots of the MTF obtained with the three denoising algorithms. All three sinogram denoising algorithms have led to an improvement in the spatial resolution in the reconstructed image. The proposed algorithm has resulted in a higher MTF than bilateral filtering and NL-PCA for all spatial frequencies.

### Real cone-beam projections of a rat

We obtained a fresh rat carcass from our institutional animal facility and used the same micro-CT scanner described above to obtain post mortem images of the rat thorax. Since the animal was obtained post mortem, no ethical approvals were required for the micro-CT scans. Because the internal organs of the rat constantly moved, we were unable to create two identical scans with different noise levels as we did for the phantom. Therefore, we scanned the rat only once. The scan consisted of 720 projections between 0° and 360° at 0.5° intervals with the tube voltage, tube current, and exposure time equal to 70 kV, 32 mA, and 16 ms, respectively.

Similar to our approach in the experiment with the physical phantom, since we do not have access to the true projections, we evaluate the performance of the denoising algorithms in terms of the quality of the reconstructed images. To create a high-quality reference image from the full set of 720 projections, we first reconstructed an initial image using the FDK algorithm. Then, we used five iterations of MFISTA algorithm [38] to improve the quality of the FDK-reconstructed image. The resulting image had a very high quality and we used it as the reference image for evaluating the performance of the denoising algorithms. We applied the denoising algorithms on a subset of 240 projections of the same scan (projections at 1.5° intervals) and reconstructed the image of the rat using the FDK algorithm.

**Fig. 7:**
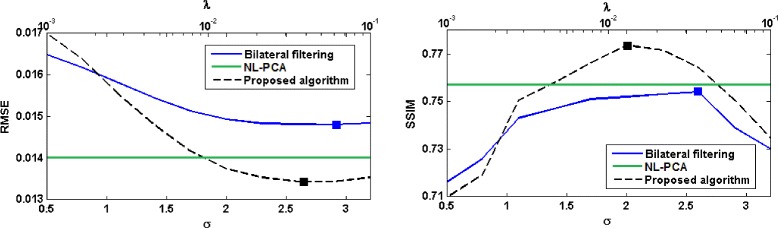
Performance comparison between different sinogram denoising algorithms on the rat scan. Values for the bilateral filtering algorithm are plotted as a function of *σ* (the *bottom horizontal axis*), whereas the values for the proposed algorithm are plotted as a function of the regularization parameter *λ* (the *top horizontal axis*). The solid squares indicate the points of optimum

Similar to the physical phantom experiment, we use RMSE and SSIM as a measure of the overall closeness of the reconstructed images to the reference image. Figure 7 shows these criteria for the three sinogram denoising algorithms. From this figure, denoising of the projections with the proposed algorithm has lead to superior results in terms of RMSE and SSIM compared to bilateral filtering and NL-PCA.

**Fig. 8:**
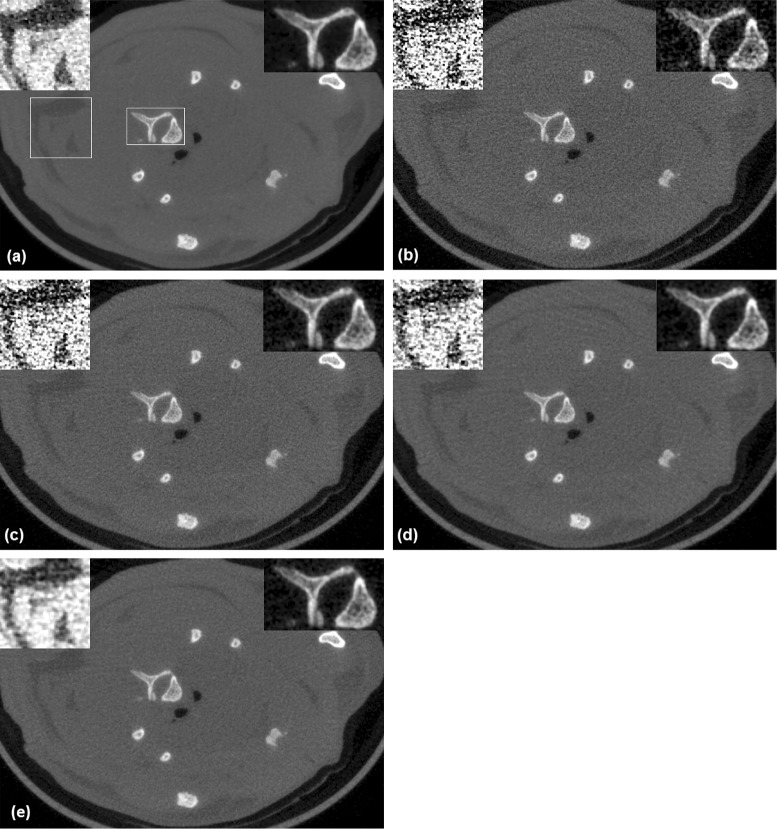
A slice of the image of the rat reconstructed from noisy and denoised projections: **a** the reference image, **b** without denoising, **c** bilateral filtering, **d** NL-PCA, and **e** the proposed algorithm. The locations of the selected ROIs have been marked on the reference image (**a**). This slice is through the thorax just below the carina (where the trachea divides into left and right bronchi)

For a visual comparison, Fig. 8 shows a typical 2D slice of the reconstructed images of the rat. For the proposed algorithm and bilateral filtering, the images shown in this figure are obtained using the parameter values that resulted in the lowest SSIM, i.e., *λ*=0.0129 and *σ*=2.6 (see Fig. 7). The window of the linear attenuation coefficient, *μ*, used to display these slices was [0,0.55]. To allow a better visual comparison, we have selected two regions of interest (ROI) within this slice and have shown them in zoomed-in views and with narrower *μ*-windows. The ROI shown on the top left of each slice contains fat surrounded with soft tissue; this ROI is shown with a magnification factor of 1.5 and with a *μ*-window of [0.15,0.20]. The ROI shown on the top right of each slice contains bone surrounded with soft tissue; this ROI is shown with a magnification factor of 2.0 and with a *μ*-window of [0.18,0.50]. These images show a strong positive effect for sinogram denoising in terms of the visual quality of the reconstructed image. Moreover, denoising with the proposed algorithm seems to have resulted in a higher-quality image, especially in the soft-tissue ROI.

**Fig. 9:**
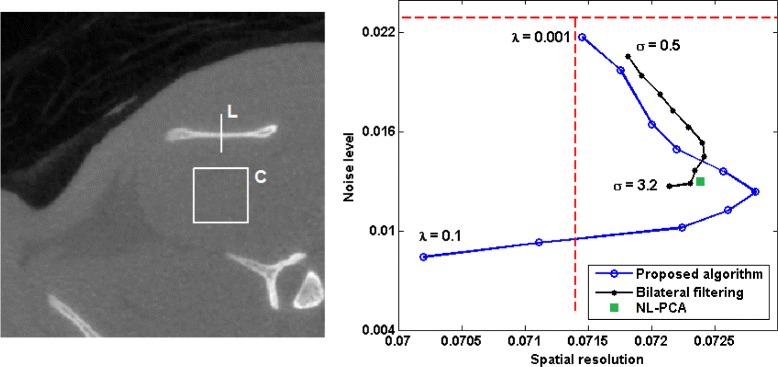
*Left*: the ROI used to compute the noise level and spatial resolution in the reconstructed images of the rat; the noise level was computed as the standard deviation of voxel values in the cube *C* and the spatial resolution was computed as the maximum gradient along the line *L*. *Right*: plots of noise level versus spatial resolution for the three denoising algorithms. The dashed horizontal and vertical lines in this plot show the corresponding values for the image reconstructed without sinogram denoising

In order to compare the denoising algorithms in terms of the trade-off between noise suppression and spatial resolution, we again followed an approach similar to that proposed in [14]. Specifically, we selected an ROI shown in Fig. 9 and computed the following measures of noise level and spatial resolution: **Measure of spatial resolution.** We compute the maximum absolute value of the gradient (i.e., slope) along the line *L* marked in the ROI shown in Fig. 9 as a measure of spatial resolution. A sharper slope indicates a higher spatial resolution and it will give a larger gradient. **Measure of noise level.** We consider a cube of size 50×50×50 voxels, the cross-section of which is shown in the displayed ROI. From the reference image, we identified this cube as being highly uniform. Therefore, we computed the standard deviation of the voxel values in this cube as a measure of noise level.

The results are plotted in Fig. 9. This plot is very similar to the plot shown for the physical phantom experiment in Fig. 6. The main observations are that all three sinogram denoising algorithms have improved the quality of the reconstructed image in terms of spatial resolution and noise level, and that the proposed algorithm can outperform the bilateral filtering algorithm and NL-PCA with the right selection of the regularization parameter. Specifically, with *λ*∈[0.0129,0.0359], the proposed algorithm has resulted in lower noise and better spatial resolution than bilateral filtering (with any choice of *σ*) and NL-PCA.

### Computation time

In order to compare the computational time of the proposed algorithm with that of bilateral filtering and NL-PCA, we considered the denoising of 240 projections of the rat. As we mentioned above, each projection was 875×568 pixels. The proposed TV-based algorithm implemented in Matlab version R2012b and executed on a Windows 7 PC with 16 GB of memory and 3.4 GHz Intel Core i7 CPU needed approximately 6 minutes to denoise all 240 projections. In comparison, bilateral filtering and NL-PCA needed 8.5 minutes and 42 minutes, respectively, for the same denoising task. In general, patch-based denoising methods such as NL-PCA require long computational times. Nevertheless, sinogram denoising methods are in general much less computationally intensive than iterative reconstruction methods. A single forward or back projection using fast algorithms such as the separable footprints algorithm [39] or the distance-driven algorithm [40] takes approximately 2 hours on the same computer; each iteration of an iterative reconstruction algorithm needs one forward-projection and one back-projection. Of course, it is becoming very common to implement iterative reconstruction methods on GPU, but the same can also be done for sinogram denoising algorithms. Sinogram denoising is by nature highly parallelizable because each projection can be denoised independently of others.

## Conclusions

Sinogram denoising can be a very effective approach to improving the quality of low-dose CT images. In this paper, we presented a fast and efficient method for denoising of low-dose CT projections. The performance of the proposed method on simulated and real CT projections shows that this method can lead to a substantial reduction in the noise level without degrading the spatial resolution. Our results show that the proposed algorithm is superior to bilateral filtering and NL-PCA in terms of denoising performance and computational speed. The proposed method is based on the assumption that the true image has a sparse gradient. Although algorithms based on this assumption have proved successful in image processing, CT projections are likely to not fit this model very well. This is because projections of piece-wise constant objects are not in general piece-wise constant, rather they are piece-wise smooth. It is well-known that TV-denoising of piece-wise smooth images can result in staircase artifacts in the denoised image. Our approach of local denoising reduces the risk of staircase artifacts. Another approach to reducing these artifacts is to consider higher-order differentials in the model. Such a formulation may lead to results comparable to those reported in this paper, but the optimization algorithms involved will be significantly more complicated. We consider this approach as a future work.

## Abbreviations

CT: Computed tomography
PCA: Principal component analysis
FBP: Filtered back-projections
ROF: Rudin-Osher-Fatemi
TV: Total variation
NL-PCA: Nonlocal principal component analysis
RMSE: Root mean square of error
MI: Mutual information
FDK: Feldkamp-Davis-Kress
SSIM: Structural similarity index
MTF: Modulation transfer function
ROI: Region of interest
